# Diphtheria Successfully Treated

**Published:** 1876-09

**Authors:** E. Chenery

**Affiliations:** Boston


### DIPHTHERIA SUCCESSFULLY TREATED.

BY E. CHENERY, M. D., BOSTON.

Everything looking toward a successful management of this so frequently fatal disease ought to be made known. This is my apology for this article, which is based on one hundred and fifty-eight cases under my care. Most of them were treated in Maine, from 1862 to 1866, and the remainder in and about Boston since that time.

I will not waste space on theories, but simply say: (1) The disease is both epidemic and contagious, and so far as the latter manner of spreading is concerned, isolation should be practiced whenever possible. (2) The disease is to be regarded not as a simply local affection, but a constitutional state, having its local expression in the throat, just as typoid fever is a general disease having its local expression in the glandular structures of the small intestines, or scarlet fever upon the skin and mucous membrane. Hence the rational indications are to deal with diphtheria as a constitutional affection rather than as local one. And (3) granting, what is generally admitted, that this constitutional state depends upon blood-poison, developed through zymotic change, the treatment, to be rational, must look to a suppression of this fermentation. The all-important question then is, Have we any means that will do it? And the object of this paper is to show that we have. I have had diphtheria myself, and so has my family, and I have treated quite a number of cases by the free use, in milk, of a tincture I have named the compound tincture of myrrh (made by digesting an ounce each of capsicum, powdered myrrh, and powdered guaiacum in a pint of alcohol), employing at the same time quinine and the tincture of iron freely, and fomenting the neck with bags of baked potatoes; but I at length came upon a case which forbade hopes from such a treatment alone. This patient was an only child, a girl of six, weakly, thin, pale, scrofulous, with tonsils well nigh meeting across the throat. Patches had formed over these, and the child was delirious.

Prof. Polli, of Italy, had broached the subject of the antizymotic powers of sulphurous acid diffused in the system. I was pleased with his statements, and felt that my time had come to make a departure in my case. I sought for the *bisulphite* of soda, and failing to get it, procured the *hyposulphite*, mixed it with syrup, and gave it to my patient in frequent doses, continuing as best I could my other remedies. Now, whatever be the value of the theory as to the action of such an agent, what interested me the most was the fact that I saved my patient.

My next patient was a little girl, daughter of a sea-captain, whose mother had come with her to visit her friends at the old homestead. At this place resided a brother of the mother, who, two years before, lost all four of his children with diphtheria, and of course they were much alarmed about this case. Believing that the hyposulphite of soda did my other patient good, and that theoretically it was adapted to meet a necessity in this class of cases, I resolved to try it again, mixing my quinine with it to save dosing. The next day my patient was better, and soon recovered.

Two girls, the only children, were taken at the same time in an adjoining house. They were treated with hyposulphite and the tincture in the same way, their throats being steamed with the potatoes. They also made prompt recoveries.

I saw in these cases what I had not seen before I began the use of the hyposulphite—a prompt suppression of the further spread of the exudation, while the patients began almost at once to improve in feeling and general appearance, and there were none of the sequelæ so sommon after this disease.

In a neighborhood two miles in another direction, there were about twenty cases coming under my care, and all got well under the hyposulphite treatment.

Though diphtheria had proved terribly fatal under other modes of management, I gradually lost my dread of it, and went from house to house treating it and seeing all my cases get well whenever they came into my hands reasonably early and my treatment was fully carried out. In two or three cases, I am sorry to say, this was not done. But in every instance of failure the fault was not due to the remedies any more than a physician is responsible for the life of the patient who has not taken the remedies he has prescribed, or is in a dying state when he is called.

One case, however, of black diphtheria, or diphtheria in which patches were of a dark color and the general look of the patient was very dusky and stupid, came under observation, and was partially treated by me. No means employed seemed to have any more effect upon the child than the surgeon's knife upon one etherized, so stolid and dead was the little patient from the very onset of the disease. This child's breath was exceedingly offensive before any patches made their appearance. I am inclined to regard this case as a singularly malignant case, over which no human skill could well be expected to have any control. And it has been the only one I have happened to see which gave evidence on the face of it that medical means would prove unavailing.

To show that it has not been my fortune to have to do with mild cases only, I will record a few instances which to my mind confirm the power which the hyposulphite of soda has over this destructive disease.

In a town twenty miles away, and past at least a dozen physi cians, the disease made its appearance, and I was informed that nearly every person who took it died. A clergyman residing there, in whose family I had practiced, made mention of me to one of his parishioners at whose house the disease had appeared, and I accordingly received a summons. The patient was a small girl with strong and thoroughly characteristic symptoms. My usual hyposulphite treatment was resorted to, and she at once began to improve. Her mother was then taken, and afterwards the clergyman's little boy and his wife. They all also recovered.

The next neighbor to the first, on the other side, had ten children and was in good circumstances. Two of the children were absent from home and one other was sent away on the outbreak of the disease, leaving seven at home. Being homœopathic in their notions, their physician was of course sent for. All of the seven took the disease and all died.

The disease broke out in a remote part of the town, and one young woman had been sick ten days, and was said to be the only one in that neighborhood to take the disease who had not died. I found her throat covered with thin patches. She was pale and very prostrate, and her friends were cautioned not to attempt to raise her up, lest the heart cease to beat. They disregarded the admonition, and she fainted, and was two hours in being restored. Five years afterward she had not fully recovered from the sickness. The poison had had an opportunity to do its work before proper treatment was applied, hence the extreme and persistent prostration. From such a state of things I fully believe my treatment would have saved her, had it been employed in time. My call, however, was to two of her sisters who were taken with the disease, having violent symptoms. For them the hyposulphite treatment was resorted to in season and the good effects promptly secured, so that they recovered without delay.

In still another neighborhood in the same town was a family of eleven, all of whom had diphtheria except the father. Three of the number had died before I was called. The fourth case was put on the use of the hyposulphite, and got well. The fifth and sixth cases were not treated by me, and died. The seventh was the mother, who was treated with hyposulphite. Her case was not bad. The eighth case was that of an emaciated married daughter of about eighteen, lately confined and so prostrate that it was with difficulty she could cross the room. Her child had died, and I was fearful that we could not save her if she contracted diphtheria. She did so, however, and was promptly put on the treatment by the hyposulphite and other measures, and came out, only rallying more slowly than others who had more strength when they were taken. Her husband then followed with the disease in a most thorough way, but the symptoms readily yielded to the hyposulphite.

There was a boy of sixteen away on the water, fishing, but soon expected home. I charged his parents as they valued his life not to let him come near the house, but on my last visit to the brother-in-law I found him sitting in the room, and when I told him I was sorry to see him there, he replid, "I'm not afraid of diphtheria." A week from that time he was taken down. The neighboring physician was called in, and not being able to check the disease, the exudation spread to the trachea and he died. Thus out of these ten cases, six died and four only recovered. All those treated by me recovered, and all those I did not treat died.

As soon as the son-in-law was able to go to his home, two miles away, he did so, taking his wife with him. Soon after, his two younger brothers took the disease at the same time, apparently having it brought to them by him. They were both thoroughly typical cases, yet yielding promptly to the same treatment that had saved the others. I might continue to multiply similar cases, but have given enough. The uniformity of the success is what tells.

A physician in a town near Boston recently lost three cases, and had another taken with worse symptoms than any of them. He lost confidence in his remedies and resolved to try mine, saying that the child could but die if he did. He saved his case, and declared to me he "never saw anything like it."

The dose of the hyposulphite is from five to fifteen grains or more in syrup, every two to four hours, according to age and circumstances. It can do no harm, but if too much is given it will physic. As much as the patient can bear without physicking is a good rule in the severer cases. The tincture can be used in doses of five drops to a half-drachm in milk. The amount for thorough stimulation is greater than can be taken in water. I usually give it in such doses as can be easily taken in milk, using the milk as food for small children. One fact, however, needs to be borne in mind, namely, the hyposulphite prevents the digestion of milk, and should not be given in less than an hour from it. They may be used alternately, however, without interference, in sufficiently frequent doses.

Judging in this disease as I judge in others, I am fully persuaded that the treatment I have so long used, and which has not failed me yet, will save nearly every case of diphtheria if seasonably and vigorously employed, and there is no reason why it should not do as well in the hands of others as in my own.

In none of my cases have I used any alcohol.

